# Editorial: Repurposed Drugs Targeting Cancer Signaling Pathways: Dissecting New Mechanism of Action Through *In Vitro* and *In Vivo* Analyses

**DOI:** 10.3389/fonc.2021.773429

**Published:** 2021-10-04

**Authors:** Eduardo López-Urrutia, Teresita Padilla-Benavides, Carlos Pérez-Plasencia, Alma D. Campos-Parra

**Affiliations:** ^1^ Laboratorio de Genómica Funcional, Unidad de Biomedicina, Facultad de Estudios Superiores Iztacala, Universidad Nacional Autónoma de México (UNAM), Tlalnepantla, Mexico; ^2^ Department of Molecular Biology and Biochemistry, Wesleyan University, Middletown, CT, United States; ^3^ Laboratorio de Genómica, Instituto Nacional de Cancerología (INCan), Mexico City, Mexico

**Keywords:** repurposed drugs, cancer, *in vitro* analysis, signaling pathways, new mechanism

## Introduction

In today’s fast-paced society, efficiency is critical in many aspects, especially in costly, resource-intensive processes such as drug discovery and development. Technological advances that expedite chemical compound synthesis or allow for parallel processing of multiple samples can only do so much to reduce the time it takes for a new drug discovered in a lab bench to reach the pharmacy aisles, mainly due to strict safety requirements that a particular drug can take years to fulfill. Enter drug repurposing, a clever strategy to reduce overall development time and cost by employing drugs already on the market –already deemed safe for consumption– and applying them to treat a disease other than it was initially approved for (1). This strategy has successfully led to the discovery of new roles for different compounds such as antibiotics (2) or analgesics (3), and is currently employed in the search for novel treatments for a wide range of conditions, from autoimmune diseases (4) to asthma (5), and even COVID-19 (6). Naturally, drug repurposing as alternative cancer therapies has become an active research area. Local and non-metastatic cancers are primarily treated with surgery and radiotherapy, while chemotherapy, hormone, and targeted therapies are currently used for advanced cases (7). However, novel treatment alternatives are needed to tackle the resistance that cancer cells develop against these drugs, while maintaining development costs under control; repurposed drugs fit the bill perfectly.

To size up research on drug repurposing, Baker and colleagues reviewed the literature relevant to drug development and found that by 2018 over 60% of the drugs used have been tested for repurposing (8). Nonetheless, reports on the efficiency of drug repurposing show success rates around 5-10% (9). This less-than-ideal efficiency calls for a switch from serendipity to purposeful analysis when searching for new repositioning candidates.

Common molecular signaling pathways contributing to the development of cancer and other diseases or conserved among different cancer types are the most conspicuous targets for drug repurposing. For instance, cyclin-dependent kinases (CDKs) exert their function in cell cycle control (10) and transcriptional regulation (11). These molecules can be targeted with anti-cancer (12) and anti-viral (13) drugs. Several groups are investigating common molecular signaling pathways in diverse cancer types, with the common goal of repurposing drugs that have been used for other diseases. Signaling pathways are not only common to various cancers; these are shared between cancer and other seemingly unrelated diseases.

**Figure 1:**
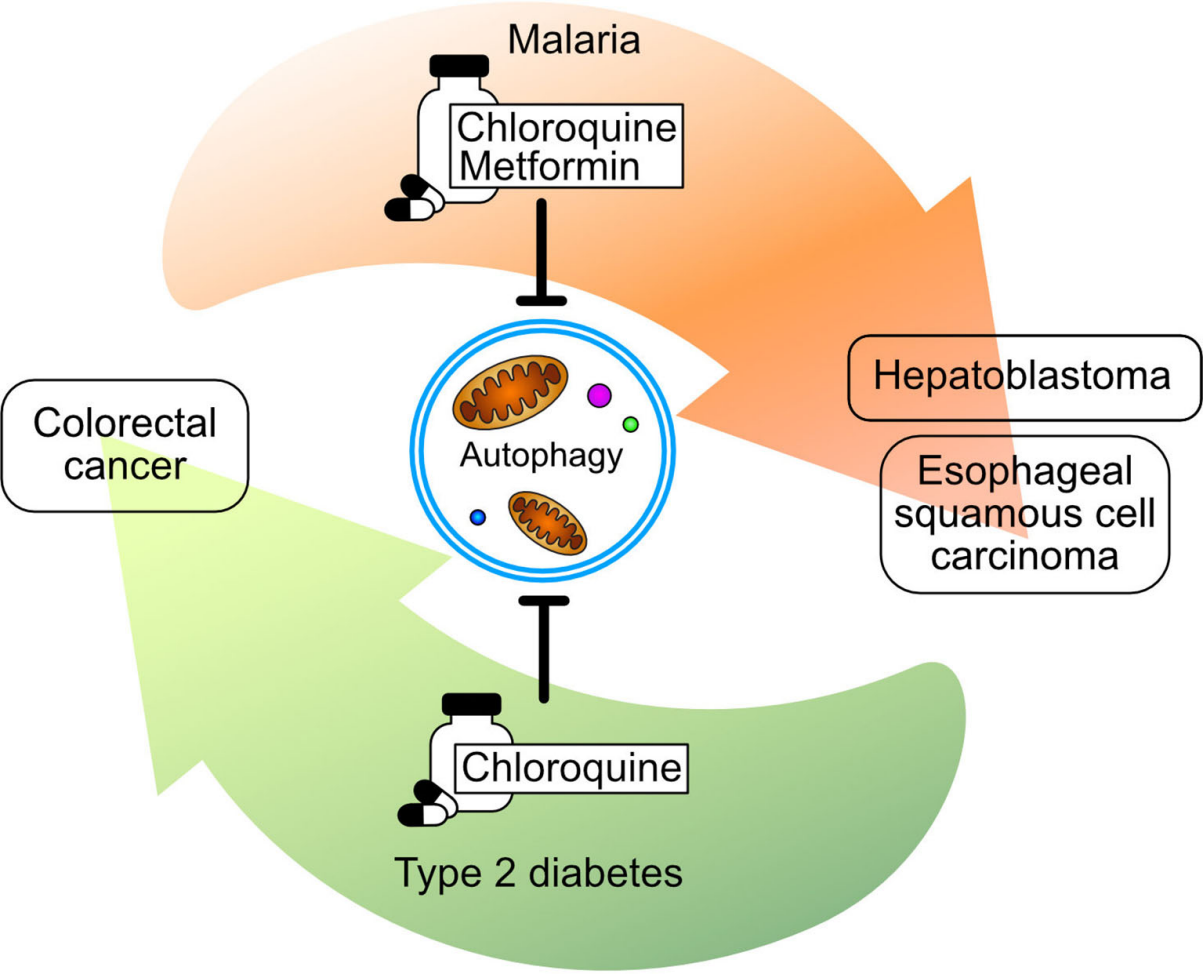
Inhibition of autophagy was a prevalent subject matter in the papers submitted to this research topic. Drugs with this effect, such as Metformin and Chloroquine, can be repurposed from malaria and type-2 diabetes to treat colorectal cancer, hepatoblastoma, and esophageal squamous cell carcinoma. Background arrows indicate the direction of drug repurposing.

This Research Topic compiled research that described drugs from all walks of pharmacology –from natural products to custom-designed molecules– that are being repositioned. Among these, *in-silico* docking analysis combined with cell and biochemical experiments demonstrated that the antifungal tioconazole can inhibit the autophagy-related protein ATG4, which decreased the viability of colorectal and breast cancer cells (14). Autophagy was further targeted by drugs originally used to treat diverse diseases such as malaria and type-2 diabetes (**Figure 1**). In this regard, chloroquine and mefloquine were originally approved as therapies against malaria, however these drugs have anticancer effects through the regulation of autophagy, as Eloranta et al. and Xie et al. demonstrated in hepatoblastoma and esophageal squamous cell carcinoma, respectively. In addition, Coronel-Hernández and collaborators combined the autophagy-regulating properties of metformin, initially approved for type 2 diabetes, and the metabolic regulation exerted by sodium oxamate with the anti-proliferating activity of a tested chemotherapy agent to achieve a synergistic effect in colorectal cancer. Finally, Xu et al. showed that the leprosy treatment clofazimine inhibits Wnt signaling impairing the growth of colorectal cancer, hepatocellular carcinoma, ovarian cancer, and glioblastoma.

Studies by Man Zhang et al., Tao Zhang et al., Wei et al., and Cheng et al. also demonstrated that anticancer drugs often target pathways common to several cancer types. Some of these are known in detail, such as apoptosis and autophagy, while others, such as LIMK1/cofilin, are less known but equally important. Active research draws a progressively more comprehensive notion of the roles of this and other novel signaling pathways in cancer, which increases the opportunities for repurposing drugs among various cancer types. However, the similarities between signaling pathways activated in different diseases alone do not guarantee drug repositioning success. Therefore, it is necessary to perform deep and purposeful analysis to ensure that these coincidences lead to successful and novel therapeutic strategies.

One of the papers in this Research Topic stood out due to its multidisciplinary approach. Xi Zhang and colleagues reported a comprehensive strategy to describe the mechanism by which anlotinib, a recently developed angiogenesis inhibitor, exerts an antimetastatic effect in pancreatic cancer. A combination of transcriptomics, proteomics, and phospho-proteomics revealed that anlotinib regulates pathways associated with endoplasmic reticulum stress, cell cycle progression, and DNA damage. The novelty in this paper resides in its search for the effects of a repurposed drug beyond known pathways into a genome-wide scenario, and of course, in its promising results. With the advent of high-throughput technologies and their increasing availability, comprehensive analyses are bound to become predominant. Undeniably, serendipity has historically had an important role in cancer drug discovery (15), as it has in science as a whole. However, the current fast-paced times demand a greater efficiency only attainable through the concerted efforts from the research community, medical practitioners, and the industry (16).

Regarding natural products, Wu et al. showed that Actein (also known as the Chinese herb “shengma”), a triterpene glycoside isolated from the rhizomes of *Cimicifuga foetida*, had a similar effect on the growth of breast cancer cells to that observed in lung cancer and osteosarcoma. These studies provided evidence of the potential broadening of application for this compound, and the importance of the continued search for natural strategies against cancer. Finally, Saavedra-Leos et al. reviewed the application of resveratrol and quercetin in combination with several nanoparticle systems to facilitate their delivery to cancer cells.

We hope that readers of this Research Topic find value in the encompassed papers and join the fascinating quest for new mechanisms of action in the realm of anticancer drug discovery.

## Author Contributions

AC-P and EL-U conceived the topic. EL-U and AC-P wrote the original draft of this editorial. All authors contributed to the article and approved the submitted version.

## Conflict of Interest

The authors declare that the editorial was conducted in the absence of any commercial or financial relationships that could be construed as a potential conflict of interest.

## Publisher’s Note

All claims expressed in this article are solely those of the authors and do not necessarily represent those of their affiliated organizations, or those of the publisher, the editors and the reviewers. Any product that may be evaluated in this article, or claim that may be made by its manufacturer, is not guaranteed or endorsed by the publisher.
